# Spatial and Temporal Scaling of Microtubules and Mitotic Spindles

**DOI:** 10.3390/cells11020248

**Published:** 2022-01-12

**Authors:** Benjamin Lacroix, Julien Dumont

**Affiliations:** 1 Centre de Recherche de Biologie Cellulaire de Montpellier (CRBM), CNRS UMR 5237, Université de Montpellier, 1919 Route de Mende, CEDEX 5, 34293 Montpellier, France; 2 Université de Paris, CNRS, Institut Jacques Monod, F-75013 Paris, France; julien.dumont@ijm.fr

**Keywords:** mitotic spindle, allometry, temporal scaling, spatial scaling, microtubule dynamics, embryonic development

## Abstract

During cell division, the mitotic spindle, a macromolecular structure primarily comprised of microtubules, drives chromosome alignment and partitioning between daughter cells. Mitotic spindles can sense cellular dimensions in order to adapt their length and mass to cell size. This scaling capacity is particularly remarkable during early embryo cleavage when cells divide rapidly in the absence of cell growth, thus leading to a reduction of cell volume at each division. Although mitotic spindle size scaling can occur over an order of magnitude in early embryos, in many species the duration of mitosis is relatively short, constant throughout early development and independent of cell size. Therefore, a key challenge for cells during embryo cleavage is not only to assemble a spindle of proper size, but also to do it in an appropriate time window which is compatible with embryo development. How spatial and temporal scaling of the mitotic spindle is achieved and coordinated with the duration of mitosis remains elusive. In this review, we will focus on the mechanisms that support mitotic spindle spatial and temporal scaling over a wide range of cell sizes and cellular contexts. We will present current models and propose alternative mechanisms allowing cells to spatially and temporally coordinate microtubule and mitotic spindle assembly.

## 1. Introduction

Metazoan development relies on biological processes that occur at very different scales, ranging from molecules to organisms, and that must be coordinated in space and time. During early embryogenesis, a succession of rapid cell divisions (embryonic cleavages) occurs in the absence of growth, leading to a dramatic reduction in cell size. How cellular processes are coordinated and remain accurate along with the progressive reduction of cell size is a fascinating question in biology. The remarkable capacity of a variety of cellular organelles to adapt their size, number or mass to the overall cell size has been the subject of many studies. Among the organelles that adapt, or scale, their size to cellular dimensions, the mitotic spindle has gathered the interest of many scientists from different disciplines for its key role in genome partitioning and its remarkable self-organizing properties. The mitotic spindle is a conserved dynamic macromolecular structure composed of microtubules essential for the alignment and segregation of chromosomes during cell division [1]. Seminal work from Peter Barlow in the 1970′s described how mitotic spindle length correlates with cellular dimensions in root meristems of maize [2]. Since this initial work, the scaling properties of the mitotic spindle have mostly been investigated in early embryos [3,4,5,6,7,8,9,10]. The progressive reduction of cell size observed across embryonic cleavage represents an ideal context to study the scaling properties of cellular organelles. In the absence of cell growth, mitotic spindles must adapt their size to the ever-reducing cell volume at each round of cleavage. This length adaptation of the spindle during early embryonic development has been observed in a wide range of organisms. Indeed, while blastomere diameters can vary from the millimeter range in frog zygotes to less than 10 µm in the blastula of most metazoans, a remarkable correlation between spindle length and cell diameter is observed across metazoans [3]. The functional requirement for efficient spindle size scaling might seem obvious, as spindle size must adapt to fit within intracellular space. However, the exact consequences of inappropriate spindle scaling are still unclear [7,9]. In physiology, allometry describes the non-linear scaling relationship between the size of an organ or an animal segment, and the body size [11,12,13]. Following pioneer biometrical analyses by naturalists of the 19th century [14], the notion of allometry (which literally means “different measure”) was introduced by Julian Huxley and Georges Teissier in their comparison of the relative increase in size of various appendages to the whole animal growth during development (namely “relative growth”, see [11,14]). The broader and most common notion of scaling is used to describe the proportionality (isometry) or disproportionality (allometry) between biological traits [12,15]. Scaling relationships between physiological traits are indeed often allometric. Their study has long been recognized for its ability to bring out underlying mechanistic principles that are responsible for the shape and size control of living organisms [11,13,16]. Similar biometrical approaches have been used at the cellular scale to analyze the scaling relationships between organelle dimensions (nucleus, cilia, mitotic spindle…) and cell size (reviewed in [15]). More recently, the relevance of this approach was extended to the study of scaling relationships at extreme scales, ranging from macromolecular assemblies to ecosystems [12]. Importantly, scaling relationships are not restricted to spatial metrics. They can also emerge from the comparison of biological traits or parameters to other dimensions such as durations. Such analyses, which take into account the time dimension, have recently led to the notion of “temporal scaling”. In biology, temporal scaling corresponds to the adaptation of the duration or rate of a biological process relative to the duration of other processes, to lifespan, or to the time span between events, such as developmental stages. For instance, the durations and developmental rates of different larval stages scale with the overall duration of animal development in *C. elegans* [17]. Temporal scaling relationships can also be observed between aging rate and lifespan in *E. coli*, *Drosophila* and *C. elegans* [18,19,20,21]. At the cellular level, temporal scaling relationships would correspond to potential correlations between the duration of organelle assembly and cell cycle length. In this line, a recent study highlighted the unexpected link between nuclear size and the duration of interphase [22]. However, if the spatial scaling of organelles, such as the mitotic spindle, has been the subject of many studies in the last decade, little is known about the temporal scaling of spindle assembly relative to various cellular, developmental and environmental contexts. Since mitotic spindles must assemble within a relatively fixed time window limited by the duration of mitosis, understanding temporal scaling in this context is particularly important. This is especially true during the successive rapid divisions of early embryos, which must be coordinated precisely to ensure proper embryo patterning.

In this review, we have summarized recent work on the mechanisms allowing mitotic spindle assembly to be coordinated in time with cell cycle events, and in space with the changes in cell dimensions. Based on the current knowledge of spindle assembly mechanisms and scaling, we propose potential scenarios for the spindle adaptation to spatial and temporal constraints imposed during rapid embryonic cleavages. Finally, we highlight some experimental observations that might be indicative of the physiological relevance of mitotic spindle spatial scaling and the temporal control of spindle assembly.

## 2. Mitotic Spindle Assembly

**Figure 1:**
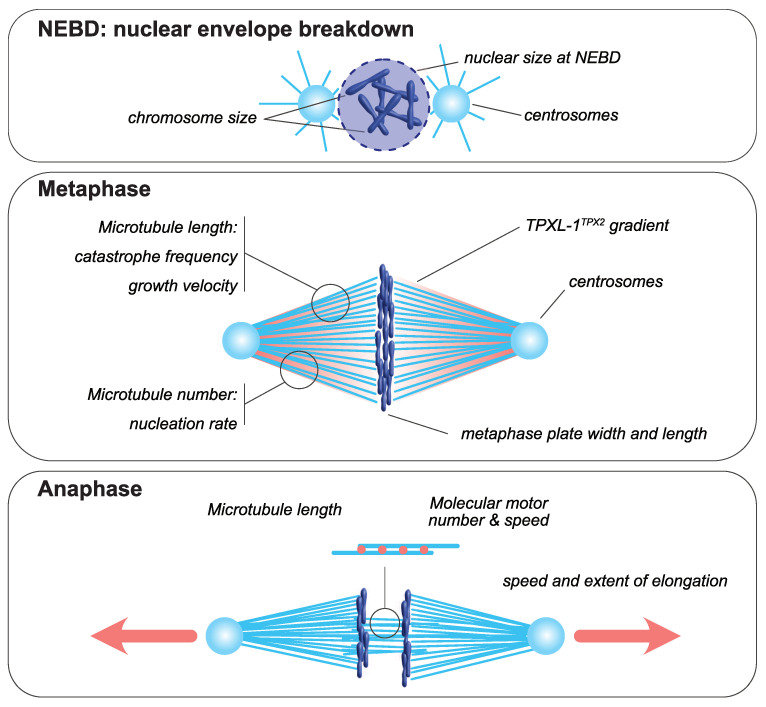
Mitotic spindle components that scale with cell size. At nuclear envelope breakdown, (NEBD, upper panel), chromosome dimensions [30,31] and the size of the nucleus scale with cell size [32,33,34,35,36]. In metaphase (middle panel), the dimensions of the metaphase plate adapt to cellular dimensions [6,31,37]. Centrosome size and a TPXL-1^TPX2^ gradient scale with cell size [4,38]. The microtubule number, through autocatalytic amplification, scales with the surface area-to-volume ratio to set spindle mass [8]. Spindle microtubule length scales with cell size and is adapted to spindle length [39]. This is achieved by changes in catastrophe frequency [9] and by the scaling of microtubule growth velocity [7,8]. During anaphase (bottom panel), the speed and extent of spindle elongation both scale with cell dimensions [5,40,41].

The mitotic spindle is a bipolar structure mainly composed of microtubules that assembles around chromosomes and orchestrates their equal partitioning between daughter cells (Figure 1). The poles of the mitotic spindle are most of the time organized around two microtubule-organizing centers (MTOCs): the centrosomes in most animal cells and the spindle pole bodies in yeast. At mitotic entry, the interphasic microtubule network is depolymerized and microtubule turnover increases [23,24]. During mitosis, microtubules nucleated at centrosomes [25,26] form two aster-like structures from which emanate astral microtubules directed toward the cell cortex. The two centrosomes are positioned around the rupturing nucleus by a microtubule-dependent process [27]. Concomitantly, a population of microtubules, hereafter termed spindle microtubules, contact and align chromosomes at the metaphase plate. Mitotic spindle assembly largely relies on the ability of microtubules to continuously and stochastically oscillate between phases of polymerization and depolymerization, a process called dynamic instability [28]. The size of the mitotic spindle is directly linked to the properties and the size of each of its individual components, including microtubules, centrosomes and chromosomes (reviewed in [29]). Analyzing how the size and assembly timing of these components is controlled during cell division is thus key to understanding the principles of spatial and temporal scaling of the spindle.

## 3. Mechanisms Ensuring Spatial and Temporal Scaling of the Mitotic Spindle

### 3.1. Microtubule Motors Enable Scaling of Spindle Elongation Rate and Duration during Anaphase

Self-organization of the mitotic spindle is driven by molecular motors that bind and slide along microtubule tracks [42,43,44]. Although microtubule motors can impact mitotic spindle length, their role in spindle length scaling during metaphase has not been reported, and several studies support the idea that molecular motors sliding activity primarily influences spindle shape and organization rather than length [45,46,47,48,49]. This is likely due to the high microtubule turnover in metaphase. During this stage, newly formed microtubules disassemble before associated motors can “sense” their length or the cell volume [50,51]. During anaphase, however, the microtubule turnover is reduced down to dynamics that become compatible with motor-dependent microtubule-length-sensing mechanisms [52,53]. In *Schizosaccharomyces pombe*, for example, the velocity of spindle elongation during anaphase is proportional to the amount of kinesin-6 Klp9, which increases with cell size [40]. This velocity indeed depends on the number of Klp9 motors bound to midzone microtubules, which is itself directly proportional to the amount of available motors and to the density and length of midzone microtubules. In a feedback mechanism, Klp9 also acts on midzone microtubule length by tuning their growth speed [41]. This makes the duration of spindle elongation and of mitosis constant in this system, and importantly, independent of initial spindle length. Therefore, Klp9 coordinates midzone microtubule sliding and elongation in a cell size-dependent manner, which ensures flawless anaphase midzone elongation with a constant duration. Whether a similar motor-dependent midzone elongation scaling mechanism exists in embryos remains to be explored. In *C. elegans* embryos, the amplitude and rate of spindle elongation during anaphase scale linearly with spindle and cell size [5]. This allows the extent of chromosome separation to be proportional to cell size, which is essential for efficient karyo- and cytokinesis. During *C. elegans* embryonic cleavages, the durations of mitosis and of mitotic spindle assembly are short and constant [7,54,55]. Thus, it is likely that the duration of anaphase is also constant throughout early embryonic divisions in this system. The scaling of spindle elongation rate in *C. elegans* embryos could, as in *S. pombe* mitosis, contribute to the temporal control of anaphase by maintaining anaphase duration independently of cell size. However, in contrast to *S. pombe*, anaphase spindle elongation in *C. elegans* does not primarily rely on midzone microtubule sliding, but rather on cortical pulling forces that are generated on astral microtubules by cortically-anchored dynein motors [56,57,58,59,60]. The amplitude of these pulling forces is proportional to the number of active force generators at the cell cortex, and to the number of astral microtubules contacting the cortex and/or to the surface area contacted by astral microtubules [56,59,61,62]. Conservation of this mechanism outside of *C. elegans* and nematodes remains to be tested, especially since, in larger embryos, astral microtubules do not necessarily reach the cortex [63]. Alternatively, pulling forces can also be exerted within the cytoplasm without any contact between microtubules and the cell cortex [64,65,66,67,68,69]. Recent in vitro assays reconstituting bulk microtubule motility have demonstrated that cytoplasmic pulling can indeed generate forces, and that the force and velocity of the movement are directly impacted by microtubule length [70]. In either scenario (cytoplasmic versus cortical pulling), the force amplitude depends on the microtubule length [71,72,73] and thus on microtubule dynamics [7,28,74,75].

### 3.2. Microtubule Dynamics Control Spindle Scaling in Space and in Time

Microtubule dynamics are generally characterized by four parameters: growth and shrinkage velocities and the frequencies of the transition between phases of growth and shrinkage, called catastrophe and rescue, respectively. These four dynamics parameters are sufficient to describe the behavior of a microtubule population in a given context [24,76]. During the cell cycle, microtubule dynamics change [52,53,77] in response to kinase activity [75,78]. In mitosis, microtubules emanate primarily from the two centrosomes and their dynamic properties change dramatically when compared to in interphase [52,78]. This drastic switch in microtubule dynamics sets an optimal average microtubule length around centrosomes, allowing for rapid and efficient chromosome capture [75]. In this high dynamics regime, called “bounded regime”, microtubule length is limited by intrinsic dynamic properties and is particularly sensitive to changes in microtubule growth velocity [75]. Therefore, modulation of microtubule dynamic properties, and thus of microtubule length, represents an efficient mechanism for controlling spindle length and the duration of assembly [7,47,79].

#### 3.2.1. Catastrophe

**Figure 2:**
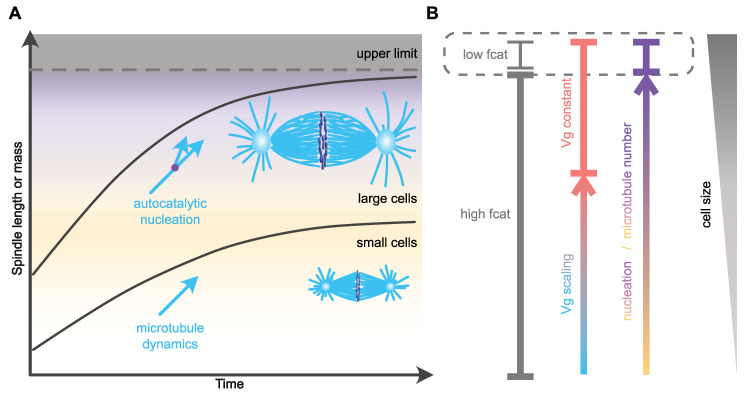
Mechanisms of spatial and temporal control of spindle assembly and scaling. (**A**) In large embryos, spindle size reaches an upper limit and does not scale with cell size [3,10]. As cell size decreases, spindle size adapts to cell dimensions through the regulation of microtubule nucleation [8]. This mechanism might allow cells to maintain the duration of spindle assembly constant. In small cells, microtubule dynamics and especially growth velocity scales with cell size and regulates microtubule and spindle length [7,8]. In *C. elegans*, this mechanism allows for the duration of spindle assembly to be constant and independent of cell size [7]. (**B**) Potential regulation of distinct microtubule parameters with cell size. The variation in catastrophe frequency (fcat) is based on observations made in *Xenopus* stage 3 and stage 8 embryo extracts, representing large- and small-cell regimes, respectively [9]. Microtubule nucleation scales over a wide range of sizes [8]. In small cells, microtubule growth velocity (Vg) scales with cell size and becomes constant in larger cells [7,8].

One of the remarkable changes in microtubule dynamics at mitotic entry is the increase in catastrophe frequency [52,75,77]. This reduction in microtubule lifetime affects microtubule length [75], and, based on in silico models, can also modulate the time of chromosome capture [79]. Thus, scaling of catastrophe frequency with cell size could represent an efficient way of controlling both size and assembly duration of the mitotic spindle. In agreement with this view, an increase in microtubule catastrophe frequency between *Xenopus* extracts prepared from stage three (four cells) and stage eight (blastula, ~4000 cells) embryos [9] was hypothesized to account for spindle length scaling in cleaving *Xenopus* embryos. The molecular mechanism proposed to control catastrophe involves a surface-area-sensing mechanism. The increase in the surface area-to-volume ratio, as cell size decreases during successive cleavages, would lead to progressive cortical sequestration of the transport factor Importin-α, through its ability to be anchored to plasma membranes. Cytosolic Importin-α can sequester and inhibit the microtubule-depolymerizing kinesin and catastrophe-inducing factor kif2a. Therefore, its progressive cortical sequestration, as cells get smaller, would in turn allow the release of kif2a into the cytosol and thus the progressive increase in the catastrophe rate [9,80]. This potential mechanism for spindle length scaling does not, however, seem to be conserved among vertebrates, and, in particular, not in zebrafish embryos or in encapsulated *Xenopus* egg extracts, where the microtubule lifetime does not vary significantly across cleavage [8]. Furthermore, alternative explanations should be considered when analyzing this result. First, caution is required when comparing extracts made from such distant stage embryos. In very large cells, mitotic spindles reach an upper-limit above which spindle length remains almost constant (Figure 2). This is the case for *X. laevis* mitotic spindle length, which is uncoupled from blastomere size during the first four embryonic divisions [10]. Then, below a given cell diameter of around 140 µm, a feature that seems astonishingly conserved across evolution [3], mitotic spindle length starts scaling linearly with cell size. This feature of spindle length scaling gave rise to the definition of two distinct regimes: the large-cell regime in which mitotic spindle length reaches a plateau and is uncoupled form cell size, and the small-cell regime of linear spindle length scaling [3,81]. Stage three and stage eight *Xenopus* embryos correspond respectively to the large- and small-cell regimes. Thus, whether the change in catastrophe frequency observed between these two stage extracts underlines mitotic spindle length scaling, or rather represents a feature of the transition point between the large- and the small-cell regimes (Figure 2), remains to be determined. Second, astral and spindle microtubule dynamic properties are distinct and vary independently across embryo cleavage [7,53]. In *C. elegans* embryos, astral, but not spindle, microtubule catastrophe frequency increases as cells get smaller [7]. The increase in catastrophe frequency between stage three and stage eight *Xenopus* egg extracts was not measured in spindles per se, but in microtubule asters nucleated from purified human centrosomes introduced in these extracts. Therefore, and potentially in line with the *C. elegans* in vivo measurements, this experimental context could highlight the behavior of astral, rather than spindle, microtubules. In this later scenario, the variation of catastrophe frequency measured in stage three and stage eight *Xenopus* embryo extracts is unlikely to affect spindle length. Indeed, a combination of experimental in vivo data in *C. elegans* and of an in silico model provided evidence that mitotic spindle length scaling is independent of astral microtubule dynamics [7,47]. Finally, an in silico model of spindle assembly [7] predicted that spindle length scaling can be recapitulated by progressively increasing catastrophe frequency, but this was accompanied by a proportional lengthening of the duration of spindle assembly as cells get smaller ([7] and our unpublished data). This result is inconsistent with the observation that mitosis duration is constant across cleavage in different species embryos. Therefore, the potential link between catastrophe frequency, mitotic spindle length scaling and assembly duration in cleaving embryos requires further investigation to specifically address its role in embryos of various size, and in both the large- and small-cell regimes.

#### 3.2.2. Growth Rate

Another microtubule dynamics parameter that can have a profound impact on spindle length in *Xenopus* egg extracts is the microtubule growth rate. Indeed, progressively raising microtubule growth velocity by adding increasing amounts of the microtubule polymerase XMAP215 in extracts leads to a proportional increase in mitotic spindle length [39]. Similar results are obtained after microinjection of XMAP215 in *Xenopus* eggs, suggesting that microtubule growth velocity could also regulate spindle length and spindle size scaling in vivo [82]. Although XMAP215 could also act on microtubule nucleation in these experiments (discussed below), modulation of the microtubule growth rate is thus a potential candidate mechanism for the regulation of microtubule and spindle length [83,84,85,86,87,88]. In agreement with this, in the nematode *C. elegans* and in the sea urchin *Paracentrotus lividus* microtubule growth rate scales with cell volume during the first rounds of embryonic cleavages [7]. In *C. elegans,* this correlation between the microtubule growth velocity and cell size does not only promote the length regulation of microtubules and mitotic spindles, it also allows for the duration of mitotic spindle assembly to remain constant and independent of cell and spindle size during cleavage. With mitotic spindle assembly duration being constant in this system, in large blastomeres the spindle assembly rate is higher than in smaller blastomeres, and linearly correlates with the average growth velocity of microtubules [7]. Thus, scaling of the microtubule growth rate with cell volume in embryos appears to be an efficient mechanism for coordinating spatial and temporal scaling of the mitotic spindle during embryonic cleavages. The mechanism by which microtubules can sense cellular volume and modulate their assembly rate accordingly is still unknown, but the limiting component model seems particularly suited [89,90,91]. According to this model, the progressive titration of positive regulators of microtubule assembly by the number of growing microtubule plus-ends could drive the proportional relationship between cell volume, microtubule growth rate and spindle length [7,82].

### 3.3. Microtubule Nucleation Controls Spindle Mass and Length Scaling in Metaphase

If spindle microtubules solely emanated from the centrosomes then, given the geometry of the spindle and the dynamic properties of microtubules in mitosis [75], their density along the spindle long axis should decrease with the distance from centrosomes. This decreasing density would inherently reduce the probability of kinetochore capture, and therefore increase mitotic spindle assembly duration as spindles get longer [79]. Thus, in large cells that assemble spindles longer than 30 µm, the number of spindle microtubules must be adjusted along the spindle length in order to maintain microtubule density [8,39,92,93]. The increase in spindle microtubule number within the spindle could also participate in a potential temporal scaling mechanism by shortening the duration of chromosome capture [79] and therefore ensuring that spindle assembly duration is uncoupled from spindle size and cell dimensions. At the molecular level, several mechanisms such as chromatin-mediated microtubule nucleation, microtubule detachment from centrosomes [10,94,95,96,97] and microtubule nucleation from pre-existing microtubules (amplification) [98,99,100,101,102,103] could account for the constant microtubule density within the spindle. Consistent with the later mechanism being an important contributor to constant microtubule density within long spindles, an in silico simulation suggested that efficient mitotic spindle assembly involves a microtubule amplification mechanism [104]. We now know that this centrosome-independent microtubule amplification, also known as the microtubule autocatalytic amplification or microtubule branching, depends on the recruitment of gamma-tubulin ring complexes (γ-TuRC) along the lattice of pre-existing microtubules by the augmin multiprotein complex [99]. The detailed molecular mechanisms behind this process of microtubule self-amplification were elegantly dissected in *Xenopus* egg extracts and found to additionally depend on the small GTPase Ran and its downstream effector, TPX2 [105]. Importantly, the exact role of this pathway in either increasing [4,106,107] or decreasing [108,109,110] spindle length is unclear, and the functional link between this pathway and mitotic spindle scaling is not fully established.

Nevertheless, recent studies in zebrafish embryos and encapsulated or cell-free *Xenopus* egg extracts have functionally linked microtubule density to spindle length, and have suggested that the number of microtubules, rather than their dynamics, is the critical parameter controlling the spatial scaling of spindles with cell size [8,106]. In *Xenopus* egg extracts, the control of microtubule number within the spindle is achieved through modulation of autocatalytic microtubule nucleation [106]. During the first divisions of zebrafish embryos, mitotic spindle size scaling occurs without any significant change in microtubule dynamics and microtubule length [8]. Instead, the decrease in spindle size is explained by a reduction of microtubule number (Figure 2). In this study, Rieckhoff et al. suggested that in large embryos, such as those of zebrafish, and following the reduction of cell size during embryonic cleavage, microtubule nucleation factors become limiting earlier than microtubule dynamics regulators. To explain this hierarchical regulation, they proposed an uncoupling between microtubule nucleation and microtubule dynamics scaling mechanisms. Using correlations between cell surface and volume, microtubule nucleation rate and dynamics, combined with mathematical modeling, they suggested that a surface area-sensing mechanism controls the scaling of the number of spindle microtubules with cell size, while microtubule dynamics and microtubule length respond to a volume-sensing mechanism [8,90,91]. The proposed molecular mechanism behind the nucleation-dependent scaling of spindle length is similar to the one hypothesized to control the catastrophe frequency in *Xenopus* embryos [9], except that a nucleation inhibitor, and not a catastrophe promoting factor is sequestered at the cell membrane. In large cells, microtubule ends would be saturated by microtubule dynamics regulators, which would account for the apparent lack of microtubule dynamics scaling. Below a critical cell size, and since microtubule number and dynamics are regulated by distinct mechanisms (surface-area sensing vs. volume sensing), the number of regulators of microtubule dynamics per microtubule would become limiting and would thus lead to the reduction of microtubule growth velocity and microtubule length. This model recapitulates remarkably well mitotic spindle scaling over a large range of cell sizes, from the upper limit down to the small-cell linear scaling regime, during zebrafish embryonic development. Overall, this hierarchical regulation model of mitotic spindle scaling suggests that microtubule nucleation, and not microtubule dynamics, is the major regulator of mitotic spindle scaling across a wide range of cell sizes. The control of microtubule dynamics would only moderately participate in spindle length modulation in small cells with a volume lying within the range of somatic cell sizes [8,92]. However, so far, neither the nature of the nucleation inhibitor, nor its mechanism of cortical sequestration, have been elucidated. Moreover, this mechanism is unlikely to be universally conserved, as mitotic spindle scaling was recapitulated in *Xenopus* extracts encapsulated in oil droplets where a membrane sequestration mechanism could not operate [8,90,91]. In these droplets, spindle scaling most likely relies on the cytoplasmic depletion of one or several limiting components [89], a mechanism that was also proposed to account for the scaling of microtubule dynamics and centrosome size [7,38,82]. Furthermore, in *C. elegans* embryos that are much larger than somatic cells, the reduction of microtubule density following the partial depletion of γ-tubulin has no effect on spindle length, and microtubule branching seems absent, with no augmin complex subunit identified so far [4]. Microtubule branching is actually not conserved in all eukaryotes [111]. Therefore, either alternative microtubule nucleation pathways are modulated in these species or microtubule nucleation is not a parameter universally controlling spindle length scaling with cell size.

Regardless of its universal conservation or not, scaling of the rate of microtubule nucleation with the cell surface also represents an efficient way of maintaining a constant spindle assembly duration independently of the final spindle size (Figure 2). Mitotic spindle architecture and geometry were suggested to be critical features influencing the timing of mitotic spindle assembly [112]. By regulating microtubule organization within the spindle, autocatalytic microtubule nucleation could in fact influence mitotic spindle architecture, and thus its assembly timing. Consistent with this view, augmin-mediated microtubule nucleation biases the directionality of microtubule growth towards chromosomes and kinetochores [105,113], which could therefore reduce the time required for chromosome capture when the centrosome-to-chromosome distance increases [79]. An important question to address is how a surface-sensing mechanism could regulate the timing of spindle assembly in very large cells, where diffusion of cytoplasmic components to the membrane would probably take longer than spindle assembly duration [81,114]. One possibility could be that the surface sensing in these large cells is, in part, regulated by active transport, which is in turn mediated by the large interphase asters [81,115]. Perturbation of these asters should thus impair the timing of the subsequent mitosis. Testing this idea will require optogenetics or physical micromanipulation approaches to perturb microtubules of interphase asters without directly affecting spindle microtubules [116].

### 3.4. Role of Other Mitotic Spindle Structural Elements in Spatial and Temporal Control of Spindle Assembly

#### 3.4.1. Nuclear Size and Initial Spindle Length

Mitotic spindle poles or centrosomes are positioned on opposite sides of the nucleus before or during nuclear envelope breakdown (NEBD). Therefore, the nucleus diameter restricts the area of spindle formation at NEBD and can thus potentially set the initial spindle length. Since the size of the nucleus is supposed to scale with cell size [32,33,34,35,36], nuclear size could in principle influence mitotic spindle size scaling by indirectly setting initial spindle length relative to cell size. However, a recent study in sea urchin and *Xenopus* embryos demonstrated that the nucleus growth rate and the duration of interphase, rather than cell size per se, defines the size of the nucleus prior to mitosis [22]. This recent study underlines an unexpected scaling relationship between the duration of interphase, the nucleus growth rate and nuclear size. It also potentially implies that the initial spindle length could scale with these three parameters. The exact relationship between initial spindle length, its final size and the duration of assembly is, however, unclear. In silico modeling of spindle assembly revealed that the time required to capture and align chromosomes is proportional to the initial nuclear radius [79]. Experimental perturbations that would specifically alter nuclear size without affecting spindle components are required to assess the potential contribution of nuclear size in setting mitotic spindle size and duration of assembly relative to cell size and to cell cycle duration.

#### 3.4.2. Mitotic Chromosomes, Kinetochores and Spindle Assembly Duration

Mitotic chromosome length scales with both cell and nuclear size during early embryonic development [30,31]. The scaling of chromosomes, which affects metaphase plate dimensions can impact spindle length or geometry [6,31,37]. In a seminal study using micromanipulation to tune the number of chromosomes in grasshopper spermatocytes, Bruce Nicklas established a link between the number of chromosomes per cell and spindle length [117]. However, later work in early embryos of various species did not confirm this view. Manipulating the DNA content in embryos can have a strong impact on spindle geometry, but only moderately affects spindle length [6,10,118,119]. However, ploidy in *C. elegans* affects spindle width [6] and should therefore have an impact on mitotic spindle volume and mass [37].

The physical connection between chromosomes and microtubules during spindle assembly is mediated by kinetochores, multi-protein complexes that assemble on chromosomes and provide an interface for microtubule attachment. In monocentric organisms that display discrete centromeres, kinetochore size does not vary proportionally to chromosome length. In contrast, in holocentric *C. elegans* embryos, where diffuse kinetochores form on the entire length of chromosomes, chromosome length scaling directly impacts kinetochore length and surface. Modulating the contact surface between kinetochores and microtubules could potentially represent an efficient mechanism to control chromosome congression and spindle assembly duration. In line with this view, in Indian muntjac fibroblasts, where chromosomes display giant monocentric kinetochores of various sizes, the number of microtubules attached to kinetochores scales with kinetochore size, leading to more efficient congression and orientation of chromosomes carrying larger kinetochores [120]. As the distance from centrosome to chromosome increases with cell size, a potential scaling of kinetochore surface with cell size could represent a way to optimize the time required for microtubules to capture chromosomes [79]. Therefore, the potential link between chromosome and kinetochore surface area and mitotic spindle size and assembly kinetics clearly needs to be further documented.

#### 3.4.3. Centrosomes and Spindle Assembly Scaling

Centrosomes are often considered as the major microtubule-organizing center in eukaryotes, especially during mitotic spindle assembly [121]. In *C. elegans* embryos, the centrosome diameter, as well as the amount of several centrosomal components essential for microtubule and mitotic spindle assembly such as γ-tubulin, also scale with cell size during embryonic development [4,38]. Centrosome scaling is thought to occur through the progressive cytoplasmic depletion of centrosomal components present in limited amount [38]. In *C. elegans* and together with centrosome size, a gradient of the microtubule-associated protein TPXL-1^TPX2^ also scales with spindle length [4]. The vertebrate ortholog TPX2, which was initially identified as the targeting protein for the motor Xklp2 to microtubules [122], was later found to be a critical regulator of the chromatin-mediated microtubule assembly downstream of the small GTPase Ran [123,124]. Independently of the Ran pathway, TPX2 also acts as a co-factor that activates Aurora A [108,125,126], a mitotic kinase essential for spindle assembly [127,128,129,130,131]. In *C. elegans* TPXL-1^TPX2^, only this later function seems to be conserved [107]. The TPXL-1 gradient, observed in *C. elegans* embryos, emanates from the centrosomes, is directed toward the chromosomes and its extent correlates with centrosome diameters, which provides a functional link between centrosome diameter and spindle length. In *C. elegans* embryos, mitotic spindle length scales with the microtubule growth rate [7], however depletion of TPXL-1^TPX2^ in *C. elegans* zygotes has a mild effect on microtubule growth velocity [53]. The link between cell size, centrosome size, microtubule dynamics and mitotic spindle scaling needs to be further documented in different systems. Whether centrosome size also scales with blastomere volume or modulates spindle length in other species embryos is unclear and would be an interesting topic for future studies.

## 4. Physiological Relevance of Spindle Scaling: A Matter of Size and/or Time?

Besides the obvious need for fitting spindle dimensions within cell boundaries, the physiological relevance of properly scaling mitotic spindle size with cell volume is unclear. A common idea emerging from the observation of mitotic spindle scaling during embryonic cleavages is that the segregating chromosomes and the resultant daughter nuclei must be sufficiently spatially distant to allow cleavage furrow formation and ingression in order to avoid cytokinesis failure [5,10,34,132,133,134]. Abnormal mitotic spindle scaling was also proposed to impact spindle positioning in *Xenopus* embryos, although without inducing any obvious defect in the embryonic cleavage pattern [9,135]. In anaphase, the extent of spindle elongation, which scales with cell size [5,40], can influence the positioning of the daughter cells within a tissue and relative to the neighboring cells. During embryonic development, this can impact the contact sites and signaling between blastomeres, and thus alter embryonic patterning [116,136]. Interestingly, an increased amplitude and speed of spindle elongation can promote the invasiveness of cancer cells in vitro [137], revealing a potential link between spindle size scaling and tumorigenesis [138]. In the same line, in tissue-cultured cells and in *Xenopus* egg extracts, inappropriate mitotic spindle size can lead to spindle pole splitting and to multipolar spindles, which often leads to chromosomal aneuploidy, a feature shared by many cancer types [118,139,140].

A critical step of mitosis or meiosis is spindle assembly, the duration of which can be experimentally shortened by inducing a higher microtubule turnover or by over-expressing the kinesin-14 HSET [141,142]. Accelerating spindle assembly through both approaches leads to increased aneuploidy in human cultured somatic cells and mouse oocytes, respectively. Conversely, a prolonged metaphase arrest is associated with increased DNA damage induced by Aurora B kinase-dependent telomere deprotection [143,144] and an elevated frequency of chromosomal nondisjunction, leading to aneuploidy [145]. The chromosomal phenotypes induced by shortening or lengthening spindle assembly duration highlight the importance of not only controlling spindle size but also the kinetics of assembly. Affecting the duration of spindle assembly might also alter the temporal coordination between chromosome segregation and other cellular events such as spindle and cell division orientation [146,147], polarity establishment [148], cortical actomyosin organization [149,150] and cytokinetic furrow ingression [151]. Such perturbations would be detrimental for cell cycle progression and development. Thus, understanding the physiological relevance of mitotic spindle scaling will require researchers to systematically consider the temporal aspect of spindle assembly and the time constraints imposed by the short duration of mitosis. The wide range of perturbations potentially associated with improper spatial and temporal scaling of the spindle also highlights the importance of studying the mechanisms that coordinate spindle scaling with cell cycle events.

## 5. Discussion

Regulation of the size of an organelle, such as the mitotic spindle, involves sizing, timing and addition mechanisms that will respectively set a certain size threshold, restrict growth by limiting its duration or systematically add a similar amount of material to a preexisting structure independently of its initial size. These mechanisms are not mutually exclusive. More recently, the notion of “folder” mechanism was proposed to account for size changes during the successive larval stages of *C. elegans*. The folder model proposes that individuals regulate either their growth rate or the duration of the developmental period to maintain a constant volume fold change [152]. Instead of a constant volume addition (adder) or a limited growth period (timer), growth rate and duration are tuned together to maintain an invariant fold change dependent on initial size. We propose that a similar mechanism operates at the cellular scale, to adapt spindle length to cell size. Indeed, in both yeast and *C. elegans* embryos, the mitotic spindle assembly rate in metaphase, its elongation rate in anaphase and microtubule growth rate all scale with cell size in order to maintain the duration of each respective process as constant and independent of cellular dimensions [5,7,40,41]. A similar principle was unraveled for cytokinesis, where the amount of contractile ring material and the speed of cytokinetic furrow ingression scale with initial cytokinetic ring diameter in both embryonic and somatic divisions allow for a constant duration of contractile ring closure [153,154].

Most described mechanisms of mitotic spindle scaling have so far aimed at explaining the regulation of final spindle length. This specific focus on length regulation, rather than kinetics or duration of assembly, is explained in part by the technical challenge posed by filming fragile and sometimes opaque embryos of various sizes. In addition, measuring mitosis duration, which is more accessible than spindle assembly duration itself, and comparing it across species may not be relevant, since each species has its own developmental rates and clocks. However, the short and constant duration of embryonic mitosis throughout early development in diverse taxa [54,55,155,156,157,158] suggests at least that a robust control of the temporality of mitotic spindle assembly is essential and deserves our attention. Combining careful descriptive analyses and quantitative approaches, coupled to mathematical and in silico modeling, will be essential to reveal the underlying mechanisms. Although not meant to be exhaustive, this review aimed to reveal how interdisciplinarity and multiscale approaches, together with the use of a wide variety of model organisms, are key to understanding the mechanisms of spindle assembly and scaling and their underlying principles.

The physiological relevance of mitotic spindle scaling has not been fully revealed so far. Studying spindle scaling in the context of early embryos could provide important insights into our understanding of embryonic development in a wide variety of contexts, including different embryo sizes, developmental timings, ecological niches or when external perturbations are applied. Aneuploidy in preimplantation embryos is usually caused by segregation errors during post-fertilization mitoses [159,160]. Thus, analyzing the causes and consequences of aneuploidy in early embryonic development, and in particular the contribution of proper spindle spatial and temporal scaling, could provide essential clues to understand and propose treatments to minimize implantation failure. More generally, understanding how mitotic spindle size adapts to cell size, and how its dimensional regulation is coupled to the temporal constraints imposed by rapid embryonic cleavages, is likely to provide substantial insights into our understanding of cell division. This could in turn contribute to developing efficient therapies that aim to control cell proliferation in pathological contexts.

## Data Availability

Not applicable.
